# Few-shot annotation correction for lightweight retinal vessel image segmentation

**DOI:** 10.3389/fmed.2026.1682878

**Published:** 2026-03-06

**Authors:** Huazhang Li, Yueming Sun, Daniel Organisciak, Yang Long, Ying Su, Feng Wang

**Affiliations:** 1 Department of Ophthalmology, Harbin Medical University, Harbin, China; 2 Hybrid-Intelligence Lab, Department of Computer Science, Durham University, Durham, United Kingdom

**Keywords:** deep learning, medical image analysis, noisy annotations, retinal vessel segmentation, U-net

## Abstract

Retinal vessel segmentation underpins quantitative analysis in ophthalmology and is widely used for screening and diagnosis. In practice, manual annotations for thin and tortuous vessels are error-prone, yet the effect of positional label noise on segmentation quality remains underexplored. We address this gap with a lightweight few-shot U-Net-based framework for annotation correction and noise robust learning. Analyses on DRIVE reveal clear performance degradation as label displacement increases. Cross-dataset validation shows that the proposed method attains an Accuracy of 96.51, an AUC of 98.01, and an F1 of 83.55 on CHASE_DB1 and an Accuracy of 97.54, an AUC of 98.45, and an F1 of 83.11 on STARE, achieving competitive performance against state-of-the-art methods. These results quantify the sensitivity of vessel segmentation to positional annotation errors and demonstrate practical robustness under noisy labels.

## 1 Introduction

Retinopathy is remarkably related to many cardiovascular and ophthalmologic diseases such as age-related macular degeneration, glaucoma, hypertension, and arteriosclerosis (1). Hemorrhages and exudates can serve as the primary distinguishing signs of retinopathy. They usually occur around the blood vessels and damage their morphological characteristics. As the retinal vascular tree is easily photographed non-invasively (2), the analysis based on retinal fundus images helps doctors identify cases. The traditional way to extract retinal vessels is manual segmentation. However, it is a laborious task that requires experienced specialists for accuracy. Developing accurate and automatic blood vessel segmentation methods is essential to realize the large-scale clinical screening of the population with retinopathy.

### 1.1 Challenges in retinal vessel segmentation

Although there have been many advanced segmentation algorithms, vascular segmentation remains a challenging task due to the limitations in obtaining medical data, the weak contrast of fundus images, and the complex geometric structure of retinal vessels. The general reasons can be summarized into the following factors:

1. Insufficient fundus images: Training deep learning models requires a large volume of data to generalize well, and a lack of data usually results in overfitting. This problem is particularly acute in shallow models, which struggle to extract high-level features necessary for accurate predictions in complex tasks, such as medical image analysis (3). Consequently, insufficient medical images have consistently posed a significant challenge in the development of reliable computer-aided diagnosis systems. Public datasets commonly used for retinal vessel segmentation, such as the DRIVE (4) dataset, which consists of only 40 images, and the STARE (5) and CHASE_DB1 (6) datasets, each containing just 20 images. These small datasets limit the ability to apply advanced data augmentation techniques or transfer learning methods effectively. The scarcity of labeled data further exacerbates this issue, making it difficult to develop robust models capable of performing well across different patient populations and imaging conditions (7).
2. Accurately segmenting retinal vessels from fundus images is a time-consuming and labor-intensive task requiring expertise from experienced clinicians. Due to the complexity involved, producing high-quality, densely annotated labels is challenging. The manual process is not only slow but also prone to variability among annotators, leading to potential inconsistencies in the labeled data (5, 8). The small size of these datasets further exacerbates the issue, as incomplete or inaccurately labeled data can hinder the performance of deep learning models (4, 6). As a result, these models may struggle to learn robust and generalizable features, complicating the development of reliable computer-aided diagnosis systems (4).
3. Indistinguishable capillaries: The weak contrast caused by poor or overexposed illumination often leads to blurred or non-sharp boundaries of blood vessels in fundus images, making it difficult for models to accurately differentiate between vessels and the surrounding tissue (8). This challenge is particularly pronounced in cases where retinal lesions are present, as these can further obscure vessel boundaries. Moreover, the width of retinal vessels varies significantly, ranging from several dozen pixels for larger vessels to just a few pixels for capillaries. This kind of fine structure increases the difficulty of the capillary recognition by models. However, the pooling layer commonly used in convolutional neural networks (CNNs) tends to remove this fine structure, which is crucial for accurate segmentation, while it is hard to rebuild during up-sampling.

### 1.2 Motivation and contribution

Most of the previous studies focused on the quantity and quality of the fundus images. They did little research on the inaccurate labeling problem. In fact, manual segmentation is error-prone and inevitably brings bias to the training of a deep learning model. Currently, some research has analyzed the effect of noisy labels on model performance (9). There are also some studies that proposed advanced algorithms to alleviate the impact of noisy labels on training (10–12). In the field of image segmentation, (13) has quantitatively studied the variation in accuracy of inaccurate annotations in the segmentation of large organs such as lungs and heart. However, the study of the impact of distorted annotations on retinal vessel segmentation tasks is still rare. In this study, we present a Few-Shot deep learning model, designed for annotation correction in lightweight retinal vessel image segmentation. This model provides advanced data-cleaning functionalities for time-saving labeling with minimal manual effort, even when working with noisy annotations. The contributions of this study include:

1. We adopt several image processing methods and design a U-Net-based model to improve the accuracy and robustness of the segmentation.
2. We apply a non-linear transformation strategy to the labels to simulate the mislabeled problem commonly encountered in real-world scenarios.
3. We quantitatively evaluate the sensitivity of the model to inaccurate retinal vessel positions and analyze the impact of these inaccuracies on segmentation performance.

Quantitative experiments substantiate that our approach effectively addresses the error-prone nature of manual retinal vessel annotations, delivering noise-robust retinal segmentation and explicitly quantifying the impact of positional label distortions on vessel topology.

## 2 Related studies

Plenty of previous studies have been done in automatic retinal vessel segmentation. With the outstanding performance that convolutional neural networks have made in image recognition tasks (14, 15), and the great success of CNN-based architectures like FCN (16) and U-Net (17) in pixel-wise segmentation, deep learning-based approaches have been widely applied to retinal vessels segmentation in recent research. The variants used by (18–22) are based on these fundamental architectures that have good performance. To reduce the spatial loss in the U-Net model, Wang et al. (21) integrated two feature refinement paths to the encoder and decoder. Their framework complemented spatial information through bilinearly up-sampling and down-sampling, and achieved high average accuracy. Liu et al. (23) introduce a multiscale U-Net augmented with spatial positional attention to better capture small vessels and boundary details, reporting improved retinal vessel segmentation on standard benchmarks. Jin et al. (22) introduced deformable convolutional blocks to a U-shaped architecture. The blocks learn receptive fields adaptively through diverse offsets and therefore model the retinal vessels of various thicknesses. In order to overcome the overfitting problem, Wang et al. (20) replaced traditional convolution with a high-frequency convolution to reduce the parameters by avoiding redundant background information. Besides, data enhancement is also an essential step. It is a common method to extract patches from images instead of training full images (8, 24, 25). In 2018, Oliveira et al. (26) proposed a rotation strategy that brought new context information to FCN. In (25, 27), a non-linear transformation had been applied to training images, which greatly enriched the input data, although it brought bias to the model.

However, noisy labels have been largely overlooked in retinal vessel segmentation, even though inaccurate annotations are common in practice. By contrast, other medical imaging areas have begun to address this issue. For example, Xiao et al. mitigate annotation noise in cardiac MRI using a dual-teacher (CNN+Transformer) semi-supervised framework with uncertainty to filter noisy pseudo-labels, and Light3DHS improves robustness to imaging noise and low contrast in 3D hippocampus MRI via a multiscale convolutional-attention encoder coupled with a lightweight ViT to enhance local–global features. Overall, explicit treatment of label noise remains rare in retinal segmentation. Zhang et al. (9) pointed out that wrong labels will seriously reduce the generalization of the model, thus resulting in the decline of accuracy. To solve this problem, some robust networks against noise have been proposed (28–30). Liu et al. (30) introduced a dual-network for medical image classification. The noise-tolerant framework can update labels from easy to difficult. Nevertheless, these effective image classification and detection approaches are not applicable to segmentation because of the additional spatial information within the images. In segmentation tasks, Zhang et al. (13) proposed a Tri-network that can mine valuable information from low-quality (non-expert) annotations, so as to counteract the effect of noise on large organ segmentation. In 2019, Shu et al. (31) proposed a spatial transformation strategy to corrode the segmentation boundary. However, due to the blood vessels' special spindly morphological feature, it cannot be applied to retinal vessel labels. In Zhou's work (32), the annotations were synthesized into the incomplete map by erasing thinner vessels directly, without considering the deviation of the position of blood vessels caused by manual segmentation. Several publicly available datasets have been commonly used for retinal vessel segmentation tasks, each offering unique characteristics that aid in advancing research.

## 3 Methodology

**Figure 1:**
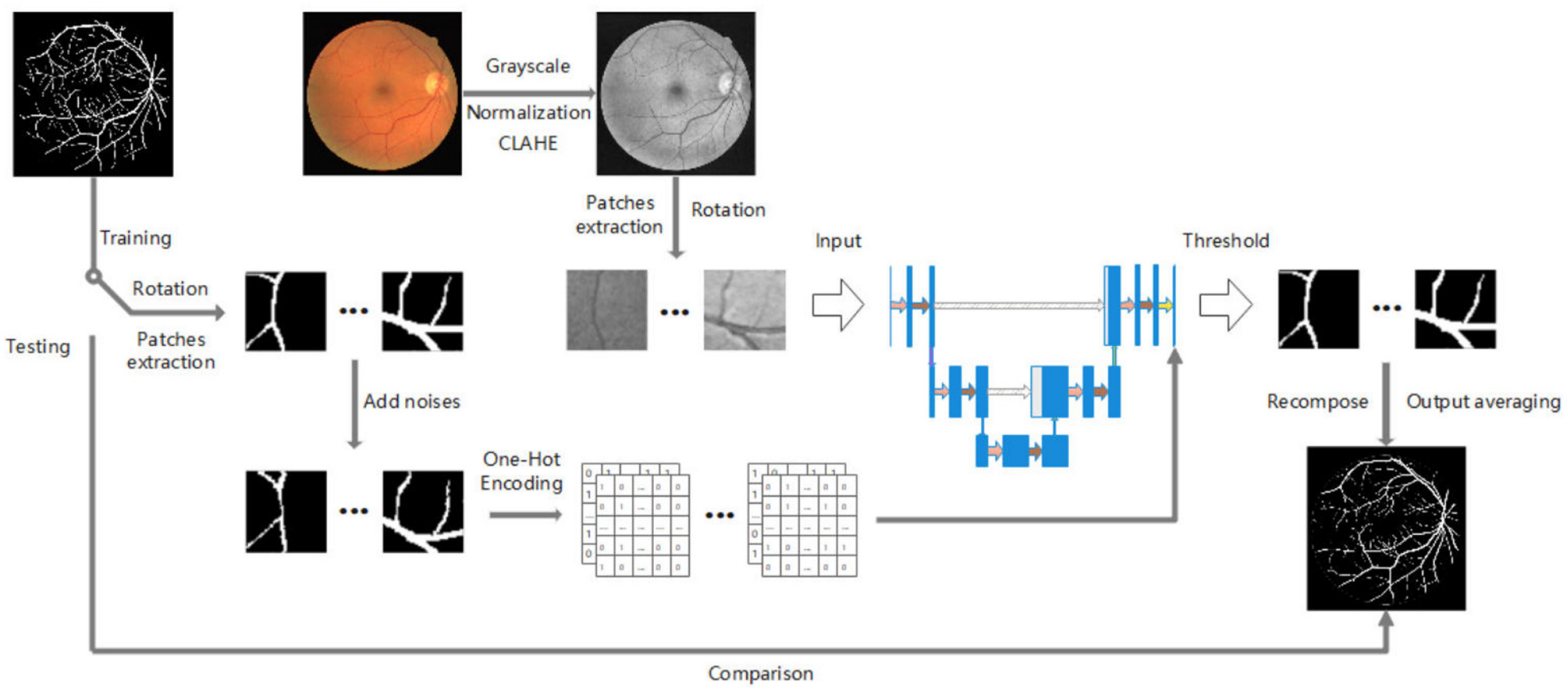
The overall pipeline of our project.

In this section, the procedure of the proposed approach is introduced in detail, including image preprocessing, patch extraction, label transformation, and model architecture. Figure 1 presents the overall pipeline of our proposed method.

### 3.1 Formalization

The algorithm outlined in Table 1 describes the dynamic attention mechanism. This framework enables the model to adaptively adjust its attention weights based on the varying input sequence *X* = [*x*_1_, *x*_2_, …, *x*_*n*_]. Inspired by this principle of dynamic, input-adaptive weighting, we propose a novel training paradigm using noise injection. We construct input sequences that intentionally intermix clear and noisy image samples. During training, the model is compelled to learn a more robust representation by dynamically attending to and distinguishing between informative clean signals and misleading noise. The objective is to train the model to develop an explicit awareness of noisy labels, thereby improving its generalization and robustness when encountering real-world, imperfect data distributions.

**Table 1:** Algorithm table of the dynamic attention framework.

**Algorithm 1: Dynamic attention framework**
1. Obtain input sequence *X* = [*x*_1_, *x*_2_, …, *x*_*n*_]
2. Compute Query *Q* from the decoder state *d*:
*Q* = *W*_*Q*_·*d*
3. Compute Key *K* and Value *V* from the encoder hidden states:
*K* = *W*_*K*_·*H*
*V* = *W*_*V*_·*H*
4. Compute the attention scores *S*:
$S=\frac{QK^{T}}{\sqrt{d_{k}}}$
5. Compute the context vector *C*:
*C* = *A*·*V*
6. Concatenate context vector *C* with the decoder state *d*:
*z* = [*C*; *d*]
7. Pass *z* through a final linear layer to generate output:
output = *W*_*o*_·*z*
8. Update the decoder state *d* for the next time step and repeat from step 2

We formalize the model training and test task as below. The training set consists of visual data and annotations. Let it be represented by (*x*_1_, *y*_1_), ..., (*x*_*n*_, *y*_*n*_) ⊆ 𝒳 × 𝒴 , where the *N* is the number of training data. The $𝒳=\left[x_{nd}\right]\in {\mathbb{R}}^{N\times D}$ represents the D-dimensional feature space, while $𝒴=\left[y_{nd}\right]\in {\mathbb{R}}^{N\times D}$ share the same dimension. During the data preprocessing, the data will be transferred to ${𝒳}_{p}=\left[x_{pnd}\right]\in {\mathbb{R}}^{N_{1}\times D_{1}}$, where *N*_1_ and *D*_1_ represent the dataset size and data dimension after the preprocessing process. This progress can be represented by 𝒳_*p*_ = *A*( 𝒳 ) where *A* means the data mapping between 𝒳_*p*_ and 𝒳 . The training progress aims to learn *f*: 𝒳_*ptrain*_ → 𝒴 where the 𝒳_*ptrain*_ is the training part in 𝒳_*p*_. We can transfer it to the following Equation:


$$\begin{array}{l} minL\left({𝒳}_{ptrain}P\right) \end{array}$$ (1)

where *P* is the projection matrix, ℒ is the loss function. At the test stage, the aim is to compare the model output *f*(𝒳_*ptest*_) with annotation 𝒴_*test*_, which can be represented by *C*(*f*(𝒳_*ptest*_), 𝒴_*test*_).

As the key idea of this project is to compare the model performance in different noise levels, here we introduce noise *no*_1_, ..., *no*_*n*_ ⊆ 𝒩 into 𝒴_*train*_. Then the training labels for model will be 𝒴_𝒩_ = 𝒴_*train*_+*no*_*i*_, where *no*_*i*_ ∈ 𝒩. Which means each noise level *no*_*i*_ is related to a label set 𝒴_𝒩i_, and using each training set can learn a model *f*_*i*_: 𝒳_*ptrain*_ (with label set 𝒴_𝒩i_ → 𝒴 ). We can transfer the comparison of the model performance into *C*(*f*_*i*(𝒳_*ptest*), 𝒴_*test*).

### 3.2 Image pre-processing

**Figure 2:**
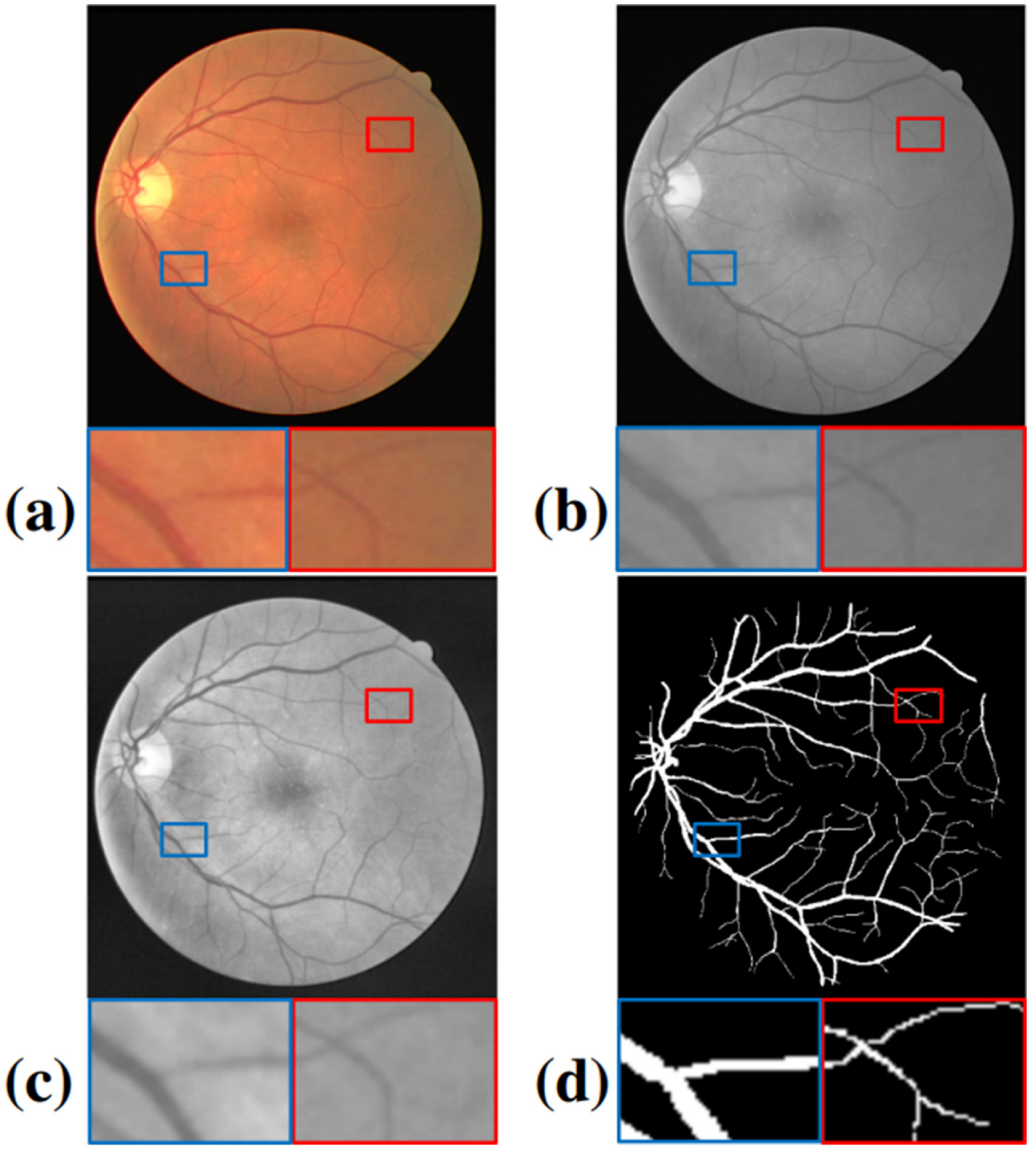
Comparison of the preprocessing results for the key steps: **(a)** Original image; **(b)** Grayscale image; **(c)** Normalization + CLAHE + Gamma adjustment; **(d)** Ground truth. The blue and red circles represent a light and dark region, respectively.

Retinal fundus images are 3-channel RGB images. To alleviate the shortage of computational resources and the adverse effects of inhomogeneous illumination intensity, the images are converted into 1-channel grayscale images, then normalized by Equation 2:


$$\begin{array}{l} I^{\prime }=\frac{I-\mu }{\sigma } \end{array}$$ (2)

where *I* and *I*′ denote, respectively, the original and converted pixel values, and μ and σ denote the mean value and standard deviation of all pixels in the grayscale images (Figure 2). Even so, the images are in low contrast, with detailed information near the edge of the retina disappearing. In this study, contrast-limited adaptive histogram equalization (CLAHE) (33) and gamma adjustment are adopted to address this issue. CLAHE can rearrange the lightness distribution according to the pixel value histograms while avoiding overamplifying noise by dividing the images into blocks. The pixel value in any range with a frequency greater than 2 will be evenly distributed among the other ranges. After the equalization, the total number of pixel values remains the same, but they are distributed more widely. This redistribution helps to enhance both the subtle features and overall contrast of the image, making finer details more apparent.

We apply Contrast-limited adaptive histogram equalization (CLAHE) to find a trade-off between vessels and noise. Specifically, CLAHE divides an image into a grid of tiles with size controlled by the tile grid parameter *t*×*t*, where *t* denotes the number of tiles along each spatial dimension. A histogram is computed and equalized independently in each tile, and bilinear interpolation is then used between adjacent tiles to suppress block boundary artifacts. To prevent the over-amplification of noise in homogeneous regions, CLAHE introduces a clip limit *c*, which caps the maximum height of each local histogram bin; the clipped excess counts are redistributed across all bins before equalization. In general, a larger *t* (smaller tiles) strengthens local enhancement and can highlight thin vessels but may amplify noise, whereas a smaller *t* yields smoother enhancement. Similarly, a larger *c* produces stronger contrast enhancement with a higher risk of noise amplification, while a smaller *c* enforces more conservative, noise-suppressed enhancement.

Gamma adjustment is one of the global tone reproduction operators (34). The operation is expressed as:


$$\begin{array}{l} J=255{\left(\frac{I}{255}\right)}^{1/\gamma } \end{array}$$ (3)

where *J* and *I* denote the original pixel values and the values after adjustment. In our experiments (shown in Table 3), γ is greater than 1, enhancing the contrast in the darker areas while reducing the contrast in the lighter areas through exponential transformation (Figure 2). This approach ensures that both bright and dark regions are well represented, which is critical for accurately visualizing retinal structures. Additionally, the combination of CLAHE and gamma adjustment provides a balanced enhancement across the entire image, making it easier to distinguish fine details such as small blood vessels and subtle pathological changes. These preprocessing steps are crucial for improving the input quality for the segmentation model, ultimately leading to more reliable and accurate segmentation results. Moreover, by standardizing the preprocessing pipeline, we ensure that the model's performance is consistent across different images, regardless of variations in lighting conditions or image quality.

### 3.3 Patch extraction

Deep learning models usually have poor performance on small datasets. They quickly tend to overfit because they are unable to learn enough features. Training with patches instead of full images brings more information to the model. Oliveira et al. (26) mentioned that more patches in a larger image benefit the model. In this study, there are 2200 overlapping patches extracted from each image during training. To balance global and local information, the size of the patch is set to 48 × 48 through experience. Besides, each patch is rotated by the angle of 90°, 180°, and 270° respectively. By doing so, the input data has been tremendously enlarged. In addition, all the pixels in the patch are guaranteed inside the FOV.

**Figure 3:**
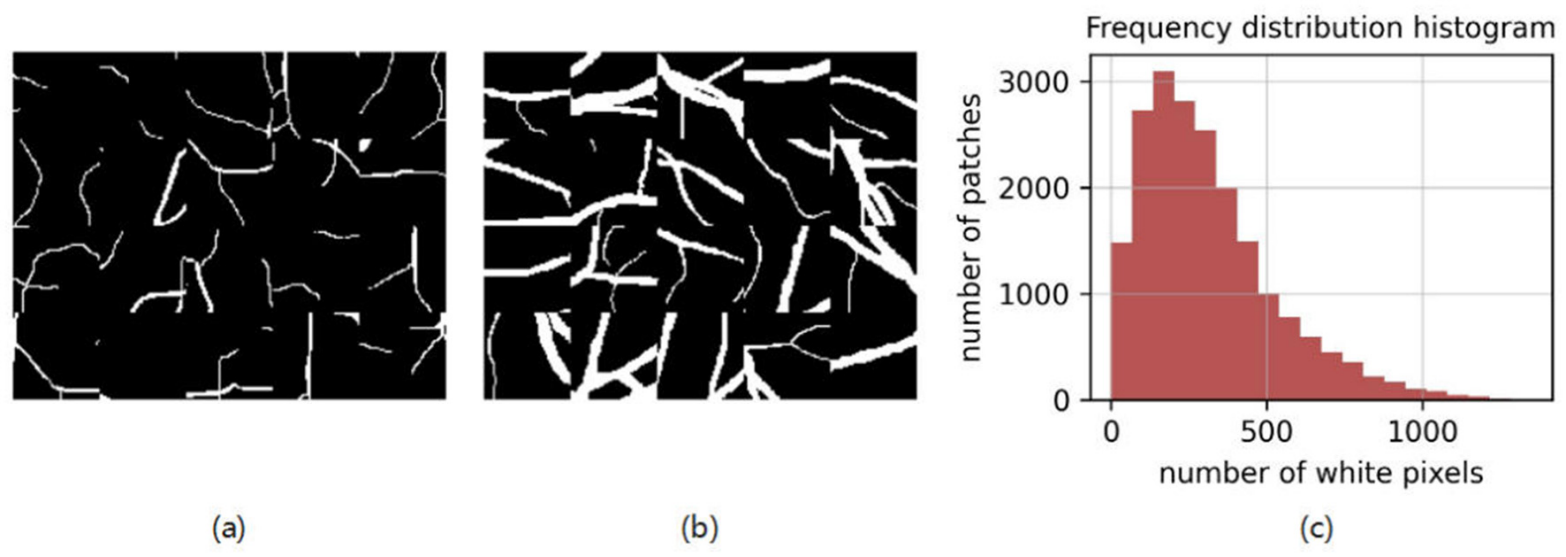
The pixel value distribution and sample patches: **(a)** Sample patches containing 0–200 white pixels; **(b)** Sample patches containing 200–800 white pixels; **(c)** The frequency distribution histogram of the number of white pixels of 1,000 randomly extracted patches.

In the retinal vessel segmentation tasks, the low accuracy of the prediction is mainly due to the insufficient segmentation ability on small blood vessels. In order to ease the problem, we randomly extract 1,000 patches from each image and count the number of white pixels (pixel value = 1) in each patch. The frequency distribution histogram is shown in Figure 3c. It has 20 bins and a heavy tail. We also visualize the patches having different ranges of white pixels, and empirically treat the patches with 0–200 white pixels as the patches containing capillary and small vessels. The proportion of small vessels to all vessels is 0.37, calculated by the first three bins of the histogram. It is plausible to believe that the data is imbalanced between small vessels and thick vessels. Therefore, to increase the extraction proportion of small vessels, assuming that the patches with a ratio of *P* of total patches are randomly selected, and the rest are only extracted from patches with 0-200 white pixels. Then the proportion of small vessels is roughly increased to 0.5. It can be expressed as Equation 4.


$$\begin{array}{l} 0.37P+\left(1-P\right)\approx 0.5 \end{array}$$ (4)

*P* is 0.8 after calculation. Thus, in our experiments, we randomly extract 80% patches, and the remaining 20% only contain small vessels.

In the test, test images are cut into patches. The usual way to do this is to seamlessly cut the image from left to right, top to bottom, in order. However, in the work by Liu et al. (35), the authors pointed out that seamless cut brings bias when combining segmentation maps of patches into the full segmentation masks. Here, we adopt their approach to cut the overlapping patches with a stride of 10 pixels in both height and width, which means most of the pixels in the image are predicted multiple times. We provide the sample patchs in Figure 3. The final results are the average of multiple predictions. This also further improves the segmentation performance.

### 3.4 Noise injection

**Figure 4:**
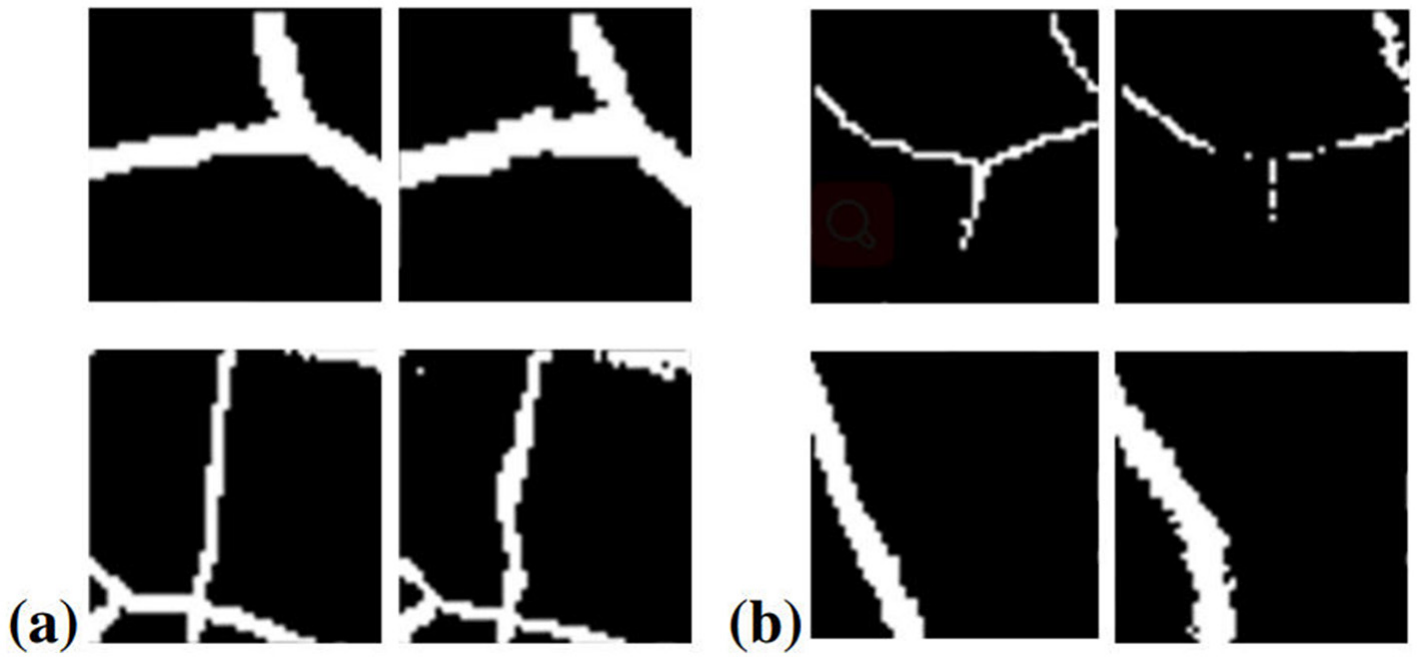
Sample transformed patches showing the effects of different β values. β = 1.5: The left pictures **(a)** denote ground truth; the right pictures **(b)** denote transformed patches.

Unlike eroding and expanding the segmentation boundary as in Zhang et al. (13) and Shu et al. (31), or directly erasing the small branches of the vessel segments in Zhou et al. (32), in our study, the method used to add noise to the labels is a non-linear transformation, which changes the position of blood vessels in the segmentation. Compared to applying traditional noise injection methods (shown in Figure 4), our noise injection strategy explicitly mimics manual annotation errors, enabling the model to learn in a more targeted manner. Every patch extracted from ground truth is transformed on a pixel level, simulating the inaccurate labeling problem existing in medical datasets, especially the errors caused by manual labeling.

Formally, let a patch be defined on coordinates (*x, y*) with center **c** = (*c*_*x*_, *c*_*y*_) and radius *R* (set to half of the shortest side of the patch, i.e., *R* = 24 in our experiments). For each pixel **p** = (*x, y*), we compute its distance to the center


$$\begin{array}{l} r=||\text{p}-\text{c}{||}_{2},\;r_{\text{norm}}=\frac{r}{R}, \end{array}$$ (5)

and apply a non-linear radial transformation


$$\begin{array}{l} r^{\prime }=Rr_{\text{norm}}^{\beta }, \end{array}$$ (6)

where β controls the deformation strength. When β > 1, the central region is effectively zoomed in; when β < 1, it is zoomed out. The new coordinate **p**′ = (*x*′, *y*′) is then obtained by


$$\begin{array}{l} {\text{p}}^{\prime }=\text{c}+\frac{r^{\prime }}{r+\varepsilon }\left(\text{p}-\text{c}\right), \end{array}$$ (7)

for pixels satisfying *r* ≤ *R*, and **p**′ = **p** otherwise, where ε is a small constant to avoid division by zero. In practice, we compute two 2D offset matrices Δ*x* = *x*′−*x* and Δ*y* = *y*′−*y* corresponding to the horizontal and vertical displacements of each pixel, and obtain the noisy label patch by sampling the original ground-truth mask at locations (*x*′, *y*′).

**Figure 5:**
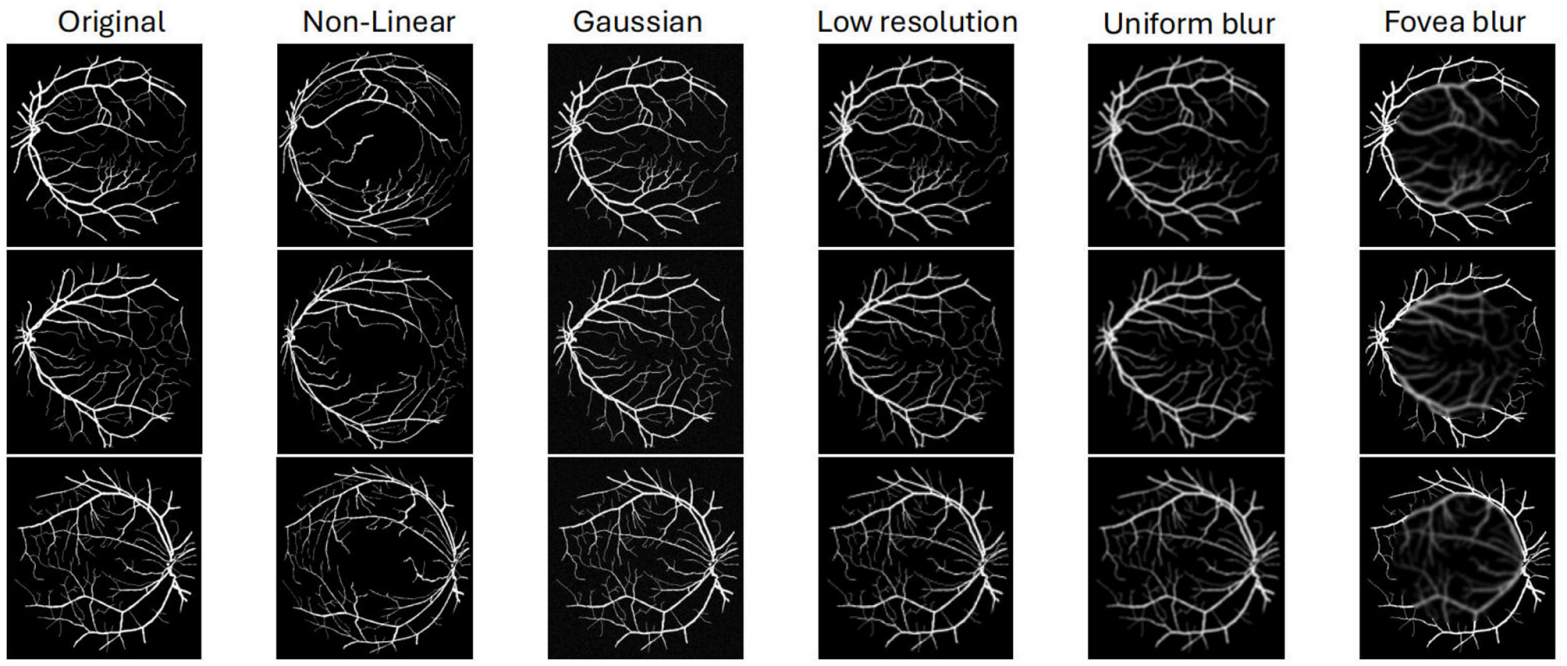
Illustration of various noise injection methods applied to the clear image. The corruptions (Non-linear, Gaussian noise, Low resolution, Uniform blur, Fovea blur) degrade image quality by introducing noise, lowering resolution, or simulating manual annotation.

In Table 2 we provide overall noise injection pipeline. The transformation region is thus a circle in the center of the patch, where the deformation ability in the central area is strongest and decreases when radiating outward. This approach allows for a more realistic simulation of annotation errors by introducing positional shifts rather than simply modifying pixel values. It preserves the structural integrity of the vessel shapes and maintains the visual context of the vessels within the retinal image. Additionally, this type of noise injection ensures that the resulting noisy labels still resemble plausible anatomical structures, which is crucial for effective model training. Some sample results are illustrated in Figure 5.

**Table 2:** Procedure of label noise injection and overlapping patch output recombination.

**Algorithm 2: Noise injection and output recombination**
1. Extract overlapping patches {*I*_*u, v*_} from *I* with size *s* and stride *t* (within *M*_FOV_)
2. Extract corresponding label patches {*Y*_*u, v*_} from *Y* using the same coordinates
3. Noise injection (labels): for each label patch *Y*_*u, v*_ and each pixel **p** = (*x, y*):
4. Let center **c** be the patch center; compute *r* = ||**p**−**c**||_2_, *r*_norm_ = *r*/*R*
5. If *r* ≤ *R*, set $r^{\prime }=R\cdot r_{\text{norm}}^{\beta }$ and ${\text{p}}^{\prime }=\text{c}+\frac{r^{\prime }}{r+\varepsilon }\left(\text{p}-\text{c}\right)$; else **p**′ = **p**
6. Obtain noisy label ${Ỹ}_{u,v}\left(x,y\right)\leftarrow Y_{u,v}\left(x^{\prime },y^{\prime }\right)$ (sample at **p**′) and one-hot encode if needed
7. Recombined probability map *P* and final segmentation Ŷ

**Table 3:** Ablation study of pre-processing parameters on retinal vessel segmentation.

**Setting**	**ACC (%)**	**F1 (%)**	**AUC (%)**
Baseline (no gamma, no CLAHE)	92.08	96.81	81.15
γ = 1.2, CLAHE (2.0, 8 × 8)	92.71	97.19	81.97
γ = 1.4, CLAHE (2.0, 8 × 8)	93.06	97.58	82.88
γ = 1.6, CLAHE (2.0, 8 × 8)	93.53	98.03	83.57
γ = 1.6, CLAHE (4.0, 8 × 8)	93.41	97.93	83.21
CLAHE, γ = 1.6 (2.0, 8 × 8)	93.18	97.69	82.96

γ denotes the gamma correction exponent, and *CLAHE*(*c, t*×*t*) indicates CLAHE with clip limit *c* and tile grid size *t*×*t*.

### 3.5 Overall pipeline

**Figure 6:**
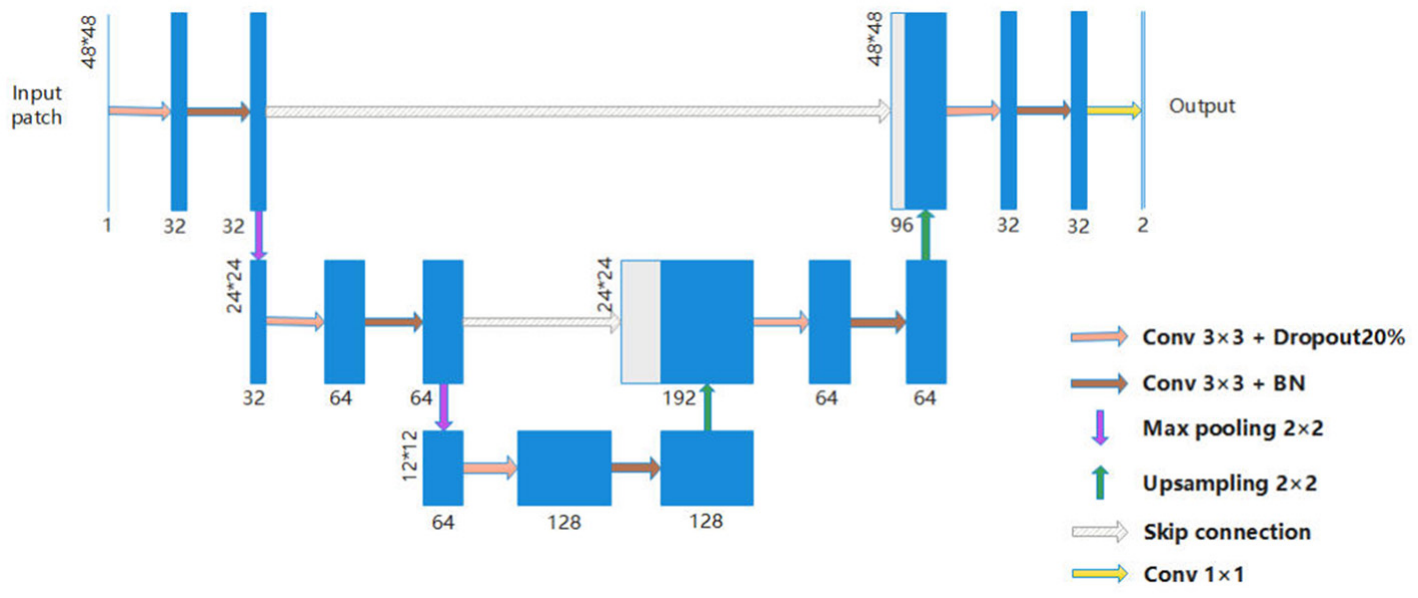
The network architecture used in our work. Blue blocks refer to multi-channel feature maps. The shape of each feature map is shown by the side of the block. Gray blocks refer to copied shallow feature maps. Arrows in different colors represent different operations.

The model is based on the U-Net proposed by Ronneberger et al. in 2015 (17). This extraordinary architecture was specially designed for biomedical image segmentation. The traditional convolutional network can extract high-level information from input images and feature maps, and reduce dimensions successfully by pooling layers, while losing detailed information. The features containing compact and positional information are challenging to reconstruct in deeper layers. However, more than recognizing objects in pictures, U-Net achieved pixel-wise segmentation by providing a symmetric structure with skip-connection, which combines both low- and high-level information. Our architecture is illustrated in Figure 6. It consists of a contracting path (on the left side) and an expanding path (on the right side). The input layer is the extracted source image and annotation. The application of two 3 × 3 convolutions repeats every step in the contracting path to summarize features from neighboring pixels, a 20% dropout layer to prevent overfitting (36). We also apply batch normalization to each step, which speeds up the training. Followed by that is a downsampling operation (max pooling layer) with a stride of 2. The size of input data in each step remains the same with the use of zero padding, and shrinks four times after max pooling, while the number of input channels is doubled. In the expanding path, the feature maps begin to be reconstructed in the same way as in the contracting path, but in the opposite direction. In every step, the input features are concatenated with the corresponding feature maps from the contracting path after 2 × 2 upsampling. The number of input channels is halved in convolutions. Finally, the dimension of the feature maps is reduced to 2 × 48 × 48 for the two-class probability in this case. The activation function used in each convolution is Rectifier Linear Unit (ReLU), and softmax is used for calculating the loss.

In our implementation, the U-Net backbone is supervised using one-hot encoded labels. For each input image patch, the encoder produces a sequence of hidden feature vectors *H* = [*h*_1_, …, *h*_*n*_] at a given scale, while the current decoder feature map at the corresponding scale is summarized into a decoder state *d*. This attention-enhanced decoder feature map is finally upsampled and passed through the output layer to predict per-pixel class logits. The corresponding ground-truth vessel masks are transformed into one-hot encodings *Y* ∈{0, 1}^*H*×*W*×*C*^ (with *C* = 2 for vessel and background), and a pixel-wise cross-entropy loss between the logits and the one-hot labels is used to train the entire U-Net with dynamic attention end-to-end.

As illustrated in Figure 1, our pipeline starts from the original color fundus images and their corresponding binary vessel annotations. The RGB fundus image is first converted to grayscale, followed by intensity normalization and CLAHE enhancement to improve vessel contrast. From the preprocessed grayscale image, overlapping patches are extracted and further augmented by rotations; these patches are then fed as input to a U-Net–like encoder–decoder network enhanced with dynamic attention. Specifically, the encoder produces multi-scale feature maps, which are passed through a dynamic attention module at the bottleneck to adaptively reweight spatial and channel responses conditioned on each input patch. In addition, before concatenation with the decoder features, the skip-connection feature maps are refined by lightweight dynamic attention blocks, allowing the model to selectively emphasize informative vessel patterns while suppressing noisy background structures. In parallel, the ground-truth vessel maps undergo the same rotation and patch extraction steps, after which artificial noise is added to the label patches to simulate noisy annotations. The noisy label patches are transformed into one-hot encodings and used to supervise the dynamically attended feature maps during training. Concretely, let ${\text{y}}_{i,j}\in {\left\{0,1\right\}}^{C}$ denote the one-hot label at pixel (*i, j*) of a patch and ${\text{p}}_{i,j}\in [0,1)C$ the corresponding softmax probability vector predicted by the network, where *C* is the number of semantic classes. We optimize the standard pixel-wise cross-entropy loss


$$\begin{array}{l} {ℒ}_{\text{CE}}=-\frac{1}{HW}\overset{H}{\underset{i=1}{\sum }}\overset{W}{\underset{j=1}{\sum }}\overset{C}{\underset{c=1}{\sum }}y_{i,j,c}\log p_{i,j,c}, \end{array}$$ (8)

where *H*×*W* is the spatial resolution of each patch. At inference time, the network outputs probability maps for each input patch; these are converted into binary vessel masks by thresholding, and the predicted patch-wise masks are recomposed into a full-size retinal vessel map, where overlapping regions are merged by output averaging to obtain the final segmentation result.

## 4 Experiment settings

### 4.1 Datasets

We consider three widely used retinal vessel benchmarks—DRIVE (37), STARE (5), and CHASE_DB1 (38)—which exhibit diverse forms of imaging and annotation noise. The DRIVE (Digital Retinal Images for Vessel Extraction) dataset consisted of 400 diabetic subjects between 25 and 90 years of age; forty photographs were randomly selected, divided into a training and a test set, both containing 20 images. The dataset was collected in a diabetic retinopathy screening program and consists of color fundus photographs acquired with a non-mydriatic 3CCD camera and stored in compressed JPEG format. This acquisition protocol introduces several sources of imaging noise: the blue channel is often nearly empty or dominated by acquisition noise, so that reliable vessel information is essentially confined to the green channel, and JPEG compression artifacts together with local illumination variations make very thin or low-contrast vessels difficult to distinguish from background texture. In addition, the ground-truth vessel masks were obtained by multiple human observers who were instructed to label pixels they judged to be vessels with at least a certain subjective confidence level; as a result, there is noticeable inter-observer disagreement, especially on subpixel-width vessels and on boundary pixels where it is ambiguous whether they belong to the vessel or to the background. These factors mean that DRIVE implicitly contains both imaging noise and annotation noise, particularly in regions of fine vasculature and near pathological structures.

The STARE (STructured Analysis of the REtina) dataset was explicitly designed to include both normal and pathological retinal images, and thus exhibits a broader and more challenging spectrum of noise and ambiguity. In abnormal cases, bright and dark lesions such as hemorrhages, exudates, drusen, and optic disc boundaries share similar local color, contrast, and curvilinear structure with blood vessels, so that a matched-filter response intended to enhance vessels also responds strongly to non-vessel structures. This leads to substantial overlap between the response distributions of vessel and non-vessel pixels and a much higher false-positive rate in the presence of pathology. Even in the hand-labeled ground truth, the authors report that pixels on vessel boundaries, very small vessels, and vessels adjacent to lesions are “less easily labeled,” and a second expert observer systematically annotates more vessel pixels than the first, reflecting genuine uncertainty in where vessels begin and end. Consequently, STARE captures both strong structured background “noise” from pathological tissue and significant inter-observer variability in the vessel labels.

The CHASE_DB1 dataset extends retinal vessel analysis to a multi-ethnic cohort of school children imaged with a hand-held fundus camera, and its images are characterized by particularly challenging noise conditions. The authors emphasize pronounced inter- and intra-image variability in background pigmentation, nonuniform background illumination, and generally poor vessel-to-background contrast, which together make small and peripheral vessels difficult to resolve. Moreover, wider arterioles often exhibit a strong central light reflex, producing a bright strip along the vessel centerline that distorts the expected Gaussian intensity profile and can confuse both human observers and automated methods attempting to delineate vessel boundaries. Compared to DRIVE and STARE, CHASE_DB1 therefore introduces additional acquisition-related artifacts specific to pediatric imaging (hand-held camera motion, pigmentation differences) and accentuates structured noise in the form of uneven illumination and central vessel reflexes, further increasing ambiguity in vessel annotation despite the availability of dual manual segmentations.

### 4.2 Training

The primary purpose of this project is to thoroughly evaluate the variation tendency of the U-Net model's performance as the proportion of noisy labels increases in the retinal vessel segmentation task, with the goal of understanding the model's robustness and the potential impact of noise on segmentation accuracy and reliability.

As described in the previous section, the main experiments are conducted on the DRIVE database. For few-shot settings, 40 photographs are randomly selected and split into 20 for training and 20 for testing. Only the results from the test set are evaluated. We also conduct cross-validation on CHASE_DB1 and STARE datasets to test generalization. To keep the same few-shot settings, we also randomly select 20 images for training and 20 images for testing. During training, 2200 patches are extracted from each image, among them 80% are randomly selected, 20% are from the region containing small vessels. Then the number of patches is quadrupled by rotation. Finally, there are a total of 176000 patches in size 48 × 48 obtained for the training set. The input annotation patches are converted to noisy labels by applying a non-linear transformation. In the five experiments, the degree of deformation is chosen in sequence of [0,0.2,0.4,0.6,0.8], where the number in the list represents the distance of *p* between non-deformation and deformation. We set 1−*p* as the noise level. The reason for doing so is that we only care about the influence brought by the displacement of the vessel from its original position, rather than the direction of transformation. Beyond that, we also conduct 3 sub-experiments for each experiment. We mix the ground truth and noisy labels in different proportions so as to further quantitatively evaluate the change in model performance with increasing noise ratio.

In the test, the output patches are regrouped to a full-size segmentation with averaging. The threshold is 0.5. Pixels with values greater than 0.5 are classified as blood vessels (1), less than 0.5 are classified as background (0).

### 4.3 Experimental setup

All the tests are conducted on a laptop equipped with an Intel Core i7-10750H CPU, an NVIDIA GeForce GTX 1650 Ti GPU, and 32GB of RAM. U-Net is used as the network model, implemented by Keras 2.3.1 with Tensorflow backend. Every model is trained for 20 epochs, with a mini-batch size of 32, using Stochastic Gradient Descent (SGD) with a 0.1 learning rate and 0.9 momentum as the optimizer. It is worth noting that the model does not stop learning after 20 epochs but tends to become very stable, demonstrating minimal improvement in performance beyond this point. Training takes about 20 min with a model size of 8.94M parameters. The choice of 20 epochs is a compromise to save training time because the segmentation task is not the main work in this study.

The training process also involves data augmentation techniques, including random rotations and shifts, to enhance the model's ability to generalize from limited training data. Each patch is augmented four times by rotating it at 90-degree intervals, effectively increasing the diversity of the dataset and reducing overfitting. This approach ensures that the model learns robust features for vessel segmentation, regardless of orientation and position within the retinal image. Model efficiency, including per-epoch training time, is summarized in Table 4. Compared with the fastest baseline, our model incurs only a modest increase in training time, while achieving the lowest parameter cost and delivering substantial performance gains (see the Results section).

**Table 4:** Model efficiency comparison: parameters, FLOPs, and inference time on different hardware.

**Model**	**Year**	**Parameters (M)**	**FLOPs (G)**	Time (CPU) (s)^*^	Time (GPU) (s)^*^
R2U-Net++	2018	4.06	63.26	1.65	1.07
UNet++	2018	3.51	29.01	1.53	1.02
Attention UNet	2018	34.88	266.53	3.55	2.59
UNet	2019	7.76	54.85	**0.97**	0.86
DU-Net	2019	4.88	53.03	1.86	1.55
NR-UNet	2023	5.72	235.92	5.10	3.58
FSG-Net	2023	10.52	44.74	1.13	0.77
DSAE-Net	2025	5.08	44.76	1.30	**0.67**
Our	2025	**3.48**	**10.61**	1.02	0.71

^*^CPU: Intel(R) Core(TM) i7-10750H CPU @ 2.60 GHz; GPU: NVIDIA GeForce GTX 1660 Ti. Bold: best; underline: second-best.

Additionally, the training is conducted with a cross-validation strategy to ensure the reliability and robustness of the model's performance across different subsets of data. The DRIVE database is split into training and validation sets, with the model's hyperparameters fine-tuned based on validation performance to prevent overfitting. This setup helps in establishing a balance between training time and model accuracy, ensuring that the results are both credible and reproducible.

Throughout the training, we monitor the loss function and accuracy on the validation set after each epoch. This monitoring helps in early stopping if the model shows signs of overfitting or if there is no significant improvement in performance, thereby optimizing the computational resources and time.

### 4.4 Evaluation metrics

We evaluate the output using several statistically significant metrics: accuracy (Acc), specificity (Sp), sensitivity (Sn), and F1 score (F1). These metrics provide a comprehensive assessment of the model's performance in the retinal vessel segmentation task, ensuring that all aspects of classification accuracy are considered. The metrics are defined as follows:


$$\begin{array}{c} Acc=\frac{TP+TN}{TP+TN+FP+FN} \end{array}$$ (9)

$$\begin{array}{c} Sp=\frac{TN}{TN+FP} \end{array}$$ (10)

$$\begin{array}{c} Sn=\frac{TP}{TP+FN} \end{array}$$ (11)

$$\begin{array}{c} Precision=\frac{TP}{TP+FP} \end{array}$$ (12)

$$\begin{array}{c} F1=2\times \frac{Precision\times Sn}{Precision+Sn} \end{array}$$ (13)

where TP, FN, TN, and FP denote true positive, false negative, true negative, and false positive, respectively. All the indicators range from 0 to 1, with higher values indicating better segmentation performance. Accuracy (Acc) reflects the overall correctness of the model's predictions, while specificity (Sp) measures the proportion of actual negatives correctly identified, which is crucial for minimizing false positives in the background. Sensitivity (Sn), or recall, indicates the model's ability to correctly identify blood vessel pixels, directly reflecting the true positive rate.

Precision measures the accuracy of the positive predictions, crucial for understanding how reliable the detected vessels are. The F1 score combines both precision and sensitivity, providing a balanced metric that is especially useful when the dataset is imbalanced, which is often the case in medical imaging. By using these metrics together, we can gain a well-rounded view of the model's performance, ensuring it accurately identifies vessels while minimizing misclassifications. Additionally, these metrics allow for the comparison of model performance across different noise levels and configurations, facilitating a thorough analysis of how well the U-Net model handles noisy labels in retinal vessel segmentation.

## 5 Results

### 5.1 Results on noise level

**Figure 7:**
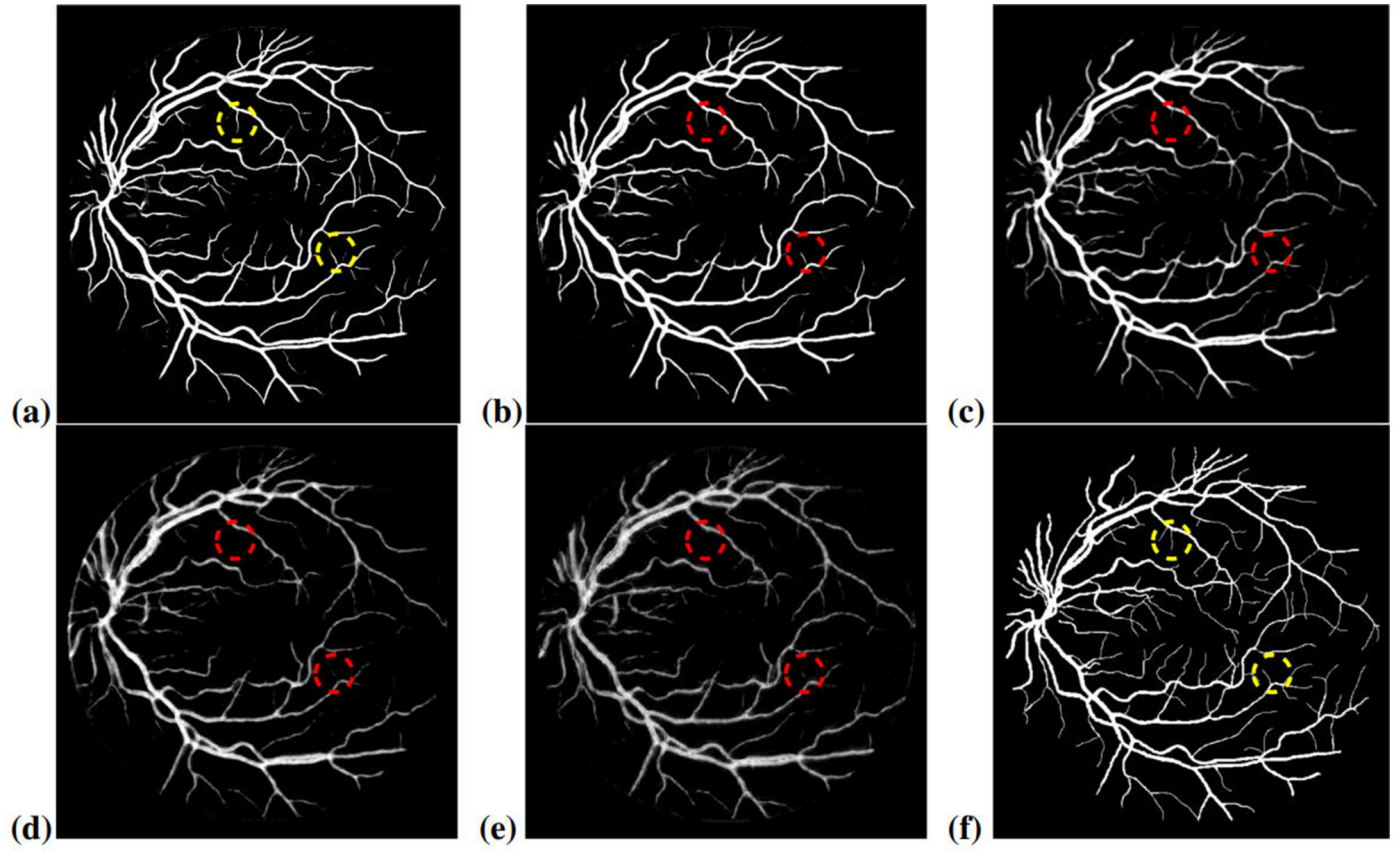
The example of segmentation results from different kinds of noise level labels: **(a)** segmentation with original labels; **(b)** segmentation with noise level lambda = 0.2; **(c)** segmentation with noise level lambda = 0.4; **(d)** segmentation with noise level lambda = 0.6; **(e)** segmentation with noise level lambda = 0.8; **(f)** ground truth. The yellow circles mark the segmented capillaries. The red circles mark the capillaries that disappear or become blurred with increasing noise level.

Table 5 illustrates the results of our main experiments. The first line gives the results generated from the model trained with original annotations. The results in the second line are based on the first model, but the test patches are extracted seamlessly (with a stride of 48), rather than overlapping patches (with a stride of 10). The third to sixth lines show the performance of segmentation with four levels of noise interference. The visual predictions are shown in Figure 7.

**Table 5:** Comparison of segmentation results with different kinds of noise levels on DRIVE dataset.

**Method**	**Acc**	**Sp**	**Sn**	**F1**
Baseline (no noise)	0.9558	0.9820	0.7758	0.8170
Baseline (seamless)	0.9498	0.9829	0.7232	0.7858
U-Net with λ = 0.2	0.9409	0.9775	0.6894	0.7479
U-Net with λ = 0.4	0.9383	0.9846	0.6215	0.7196
U-Net with λ = 0.6	0.9218	0.9897	0.4566	0.5978
U-Net with λ = 0.8	**0.9198**	**0.9919**	**0.4255**	**0.5746**

Bold indicates the best value.

In Table 5, compared with the baseline, which is on the averaging of multiple patch predictions, the use of seamless output recombination is obviously not practical. Meanwhile, the other experiments quantify the influence of inaccurate annotations on retinal vessel segmentation. Most of the indicators decline as expected when the noise level increases, except specificity. It presents a transitory fall and climbs back in the later experiments. Specificity refers to the true negative rate. The higher specificity means a lower proportion of backgrounds misclassified as blood vessels. However, compared to the 3.22% drop in accuracy, the decreasing amplitudes are dramatic in sensitivity and f1 score, being 30.95% and 22.12% respectively. Sensitivity refers to the true positive rate, and the F1 score takes both precision and sensitivity (recall) into account, which is a reliable metric to measure the performance of a classification model. The plunge of these two indicators demonstrates the severe deterioration of the network's identification ability for blood vessels after the noise level reaches 0.6. The model tends to classify pixels as backgrounds, resulting in the rise of specificity. In Figure 7, the segmentation blurs with the increasing noise due to the weaker classification probability on vessels, especially on small vessels. In the red circles, capillaries are faded away with noise interference. Most of them disappear when the noise level reaches 0.6. In Figure 7d, an abnormal edge shows on the left of the segmentation. The network mistakes the color difference between the FOV and outside as a blood vessel, which means the degradation of the model's noise-robust learning ability.

### 5.2 Results on noise types

Table 6 summarizes the influence of different noise types on retinal vessel segmentation performance. The first row reports the results obtained when training the model with our proposed non-linear positional perturbation of the vessel labels. This setting achieves the highest accuracy, F1 score and AUC, indicating that the non-linear transformation provides a challenging yet still anatomically plausible supervision signal that helps the network learn more robust vessel representations. The subsequent rows correspond to image-level corruptions applied to the fundus images: additive Gaussian noise, low-resolution downsampling, uniform blur, and fovea blur. Among these, Gaussian noise and uniform blur lead to a moderate degradation in performance, suggesting that the model can still recover most vessel structures under global intensity fluctuations and mild smoothing.

**Table 6:** Ablation study of different noise types on retinal vessel segmentation.

**Noise type**	**ACC (%)**	**F1 (%)**	**AUC (%)**
Non-linear	**93.48**	**97.98**	**83.62**
Gaussian	93.10	97.70	83.10
Low resolution	92.35	96.95	81.80
Uniform blur	92.88	97.30	82.40
Fovea blur	92.70	97.15	82.10

Bold indicates the best value.

In contrast, the low-resolution setting yields the most pronounced drop in all three metrics, reflecting that aggressive downsampling and upsampling severely damage thin and tortuous vessels and thus hinder the network's ability to localize small structures. Fovea blur also reduces performance, but to a lesser extent than the low-resolution case, as the corruption is confined to the central region and leaves peripheral vessels relatively intact. Overall, the ablation demonstrates that different noise types affect the segmentation model in distinct ways: positional label noise can be exploited to regularize training and improve robustness, whereas strong resolution degradation or localized over-blurring primarily impairs the detection of fine capillaries and leads to a more noticeable decline in F1 score and AUC.

### 5.3 Results on noise ratio

**Figure 8:**
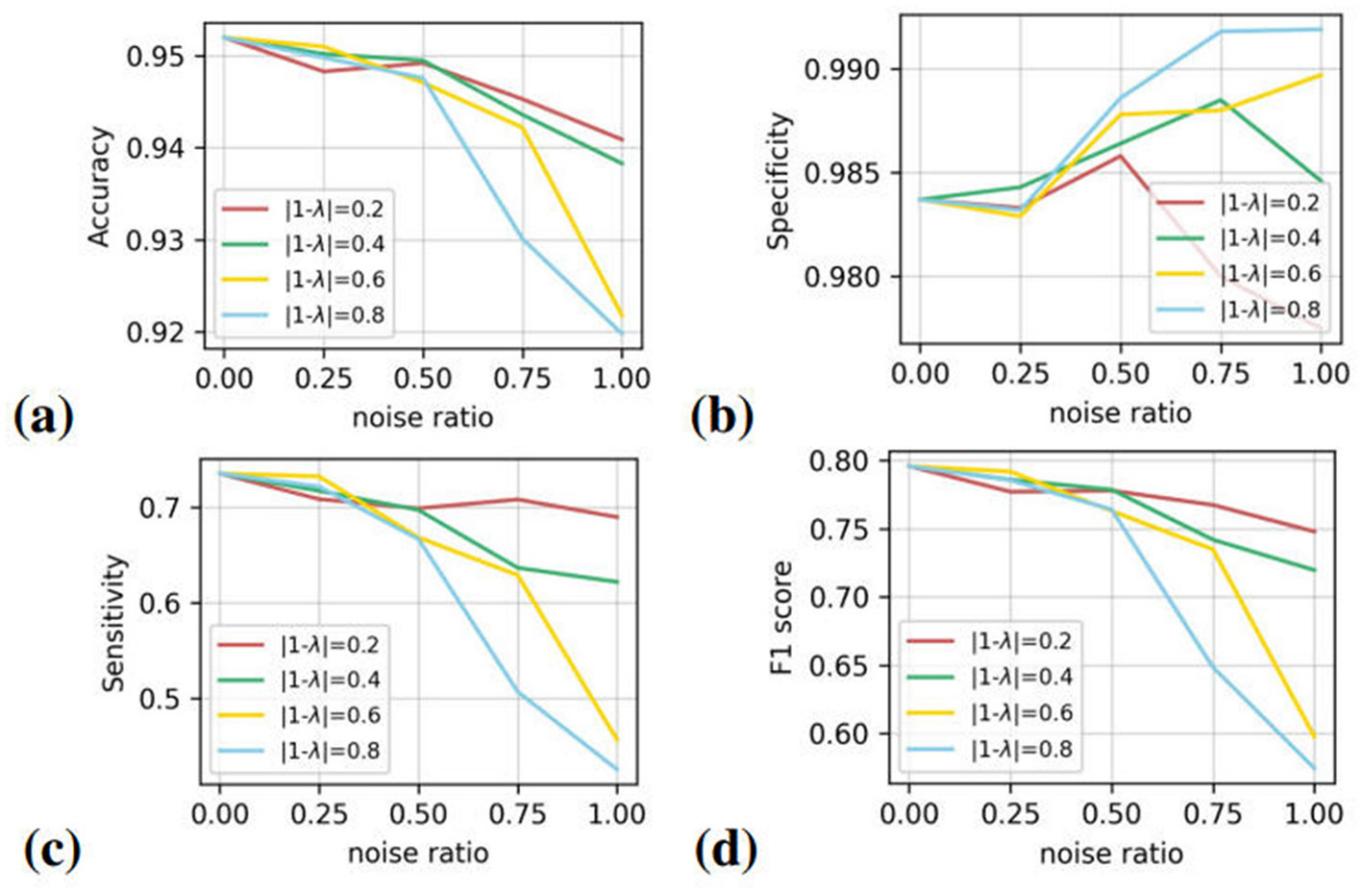
The segmentation performance under different noise levels with noise ratio at [0, 0.25, 0.5, 0.75, 1]. The figures from left to right represent four evaluation matrices. Four lines in different colors represent for noise ratios. Here, λ denotes the ratio of original images, and 1 − λ denotes the noise ratio.

As in many cases, the noises exist as part of the overall data. Here, we conduct several sub-experiments to simulate the actual situation. At every noise level, we evaluate the segmentation performance with different noise ratios at [0, 0.25, 0.5, 0.75, 1], where 0 represents original labels. The results are illustrated in Figure 8.

In the figure, when the noise ratio is less than 0.5, the indicators vary slightly. However, there are striking changes in all the indicators when more than half of the labels are noisy. The lines spread out when the x-coordinate is greater than 0.5. It can be concluded that the segmentation performance of U-Net has no linear correlation with annotation noises (in the form of position offset). Only when the noise dominates the label, the identification ability on vessels of the network is disrupted, apparently. The variation tendency of evaluation metrics is similar to that of the main experiments. The sensitivity and F1 score of the models with the noise level of 0.6 and 0.8 decrease rapidly, while the specificity has a slight rise.

### 5.4 Cross dataset validation

To assess generalization beyond DRIVE, we evaluate on CHASE_DB1 and STARE (Table 7). We keep the same split setting with the Drive dataset, a random split of 1:1 train and test datasets. Compared to the Drive dataset, the CHASE_DB1 and STARE contain no official train/test splits, so we reimplement all the compared baselines under a fixed split protocol for fair comparison. Our Proposed method achieves the best results on STARE across all three metrics—Acc 97.54%, AUC 98.45%, and F1 83.11%, and ranks second on CHASE_DB1 with Acc 96.51%, AUC 98.01%, and F1 83.55%, closely trailing the best F1 of Attention U-Net and the best Ac/AUC of DU-Net. Compared with the plain U-Net baseline, our method improves F1 by +5.72% on CHASE_DB1 and by +3.65% on STARE, with concurrent gains in Acc and AUC, respectively. Taken together with the noise-level and noise-ratio analyses, these cross-dataset outcomes indicate that the model's robustness to label perturbations translates into better vessel delineation under dataset shift, particularly on STARE, where small-vessel preservation is reflected in the consistently superior F1.

**Table 7:** Results on CHASE_DB1 and STARE.

**Methods**	**CHASE_DB1**			**STARE**		
	**Ac**	**AUC**	**F1**	**Ac**	**AUC**	**F1**
R2U-Net (19)	95.56	97.84	81.71	95.29	97.68	81.79
U-Net (22)	95.78	97.72	77.83	95.01	97.81	79.46
DU-Net (22)	**96.61**	**98.12**	80.37	96.59	98.26	80.64
Unet++ (39)	96.15	97.98	83.25	96.50	97.64	82.53
Attention U-Net (40)	96.08	97.98	**83.84**	96.37	97.79	82.36
**Proposed** **method**	96.51	98.01	83.55	**97.54**	**98.45**	**83.11**

Each dataset reports Accuracy (Ac), AUC, and F1-score (%).

Best is in Bold, second-best is underlined.

### 5.5 Evaluation of data cleaning

While we have cross-tested the sensitivity of the model to different noise ratios and levels, there is no advice on data cleaning. To verify the necessity of further noise cleaning, we conduct additional experiments on the data where noise has been removed, keeping other parameters the same. The training data with 25%, 50%, 75% noises in the above experiments are reduced to $\frac{3}{4}$, $\frac{1}{2}$ and $\frac{1}{4}$ respectively. The results of the same amount of training data are averaged. We select the most representative sensitivity and F1-score as the evaluation matrices. Experiment results are illustrated in Table 8. As the noise ratio increases and the amount of clean data decreases, our method consistently stabilizes performance. This demonstrates strong robustness to noisy annotations as well as effective learning under limited clean supervision (few-shot setting).

**Table 8:** Comparison of segmentation results with noisy and clean data on DRIVE dataset.

**Noise ratio**	**Methods**	**Noisy data**		**Label**	**Clean data**	
		**Sn**	**F1-score**		**Sn**	**F1-score**
0.25	R2U-Net (19)	0.7216	0.7853	Cleaned	0.7421	0.7980
	U-Net (22)	0.7222	0.7853	Cleaned	0.7395	0.7964
	DU-Net (22)	0.7212	0.7847	Cleaned	0.7408	0.7971
	UNet++ (39)	0.7231	0.7865	Cleaned	0.7440	0.7992
	Attention U-Net (40)	0.7212	0.7853	Cleaned	0.7453	0.8001
	NR-UNet (41)	0.7061	0.7726	Cleaned	0.7453	0.8010
	t-vMF (42)	0.7081	0.7770	Cleaned	0.7486	0.8022
	T-Loss (43)	0.7168	0.7857	Cleaned	0.7479	0.8018
	Ours	0.7319	0.7917	Cleaned	**0.7560**	**0.8077**
0.5	R2U-Net (19)	0.6487	0.7239	Cleaned	0.6618	0.7315
	U-Net (22)	0.6564	0.7321	Cleaned	0.6649	0.7332
	DU-Net (22)	0.6531	0.7314	Cleaned	0.6605	0.7326
	UNet++ (39)	0.6428	0.7246	Cleaned	0.6662	0.7360
	Attention U-Net (40)	0.6395	0.7128	Cleaned	0.6637	0.7341
	NR-UNet (41)	0.6659	0.7362	Cleaned	0.6704	0.7391
	t-vMF (42)	0.6635	0.7248	Cleaned	0.6723	0.7395
	T-Loss (43)	0.6317	0.7275	Cleaned	0.6947	0.7462
	Ours	0.7019	0.7617	Cleaned	**0.7060**	**0.7577**
0.75	R2U-Net (19)	0.5196	0.5638	Cleaned	0.5417	0.6124
	U-Net (22)	0.5241	0.5549	Cleaned	0.5483	0.6187
	DU-Net (22)	0.5162	0.5611	Cleaned	0.5379	0.6073
	UNet++ (39)	0.5037	0.5462	Cleaned	0.5448	0.6195
	Attention U-Net (40)	0.5208	0.5566	Cleaned	0.5526	0.6311
	NR-UNet (41)	0.5094	0.5381	Cleaned	0.5862	0.6554
	t-vMF (42)	0.5149	0.5367	Cleaned	0.5718	0.6423
	T-Loss (43)	0.5173	0.5592	Cleaned	0.5751	0.6486
	Ours	0.6319	0.6917	Cleaned	**0.6560**	**0.6877**

Best is in Bold, second-best is underlined.

In the table, the data in bold represent the best results in the same set of experiments. It is notable that noise-removed data perform significantly better than data with noise, although fewer data points are trained. Meanwhile, even the slightest noise interference in the experiment affects the model's determination. It will not bring additional information, though with more data. We figure that it is productive to clean the noisy data and labels in retinal vessel segmentation tasks.

## 6 Discussion

The primary goal of this study was to evaluate the impact of noisy labels on the performance of the U-Net model in retinal vessel segmentation tasks. Our experimental results provide valuable insights into how noise levels and noise ratios affect the segmentation accuracy and robustness of the model.

### 6.1 Impact of noise levels on model performance

The results from Table 5 indicate a clear degradation in model performance as the noise level increases. Specifically, as the noise level *p* rises from 0 to 0.8, we observe a significant decrease in sensitivity (Sn) and F1 score, which are critical metrics for assessing the accuracy of vessel segmentation. Sensitivity, which measures the true positive rate, plummets from 0.7758 in the baseline to 0.4255 at the highest noise level. This drop reflects the model's declining ability to correctly identify blood vessels when noise is introduced. Similarly, the F1 score, which considers both precision and recall, shows a notable reduction, indicating that the overall quality of the segmentation deteriorates with increasing noise.

Interestingly, the specificity (Sp), which measures the true negative rate, demonstrates an initial decline but then improves at higher noise levels. This rise in specificity, despite the decrease in other metrics, suggests that the model becomes overly conservative in classifying pixels as background when noise is high. As a result, fewer non-vessel pixels are misclassified as vessels, but at the cost of missing actual vessel pixels, which is detrimental to accurate segmentation.

Building on this trend, we further compare against existing noise-robust segmentation frameworks (Table 9). Among the baselines, t-vMF and T-Loss are loss functions integrated into the U-Net. t-vMF attains the highest Dice and Acc (0.8868 and 0.9052), while our proposed method yields the best IoU (0.8506), with NR-UNet close behind on Dice/IoU and T-Loss providing the second-best Acc. This pattern highlights our method's ability to handle noisy labels. Taken together, these results indicate that robustness strategies trade off different aspects of performance under noise, and our method complements the strongest baselines by prioritizing accurate region overlap when sensitivity and F1 begin to decline at higher noise levels.

**Table 9:** Comparison of segmentation results with existing noise-robust segmentation frameworks.

**Method**	**Dice**	**IoU**	**Acc**
NR-UNet (41 )	0.8703	0.8424	0.8974
t-vMF (42)	**0.8868**	0.8121	0.9052
T-Loss (43)	0.8255	0.8096	0.9045
DSAE-Net (44)	0.8244	0.7986	**0.9548**
FSG-Net (45)	0.8420	0.8173	0.9044
Proposed methods	0.8632	**0.8506**	0.9042

Dice refers to the Dice similarity coefficient and IoU denotes Intersection over Union. Best values are in Bold, and second-best are underlined.

### 6.2 Effect of noise ratio on model performance

The analysis of different noise ratios, as shown in Figure 8, reveals that the U-Net model's performance is not linearly correlated with the amount of noise in the data. When the noise ratio is below 0.5, the model maintains relatively stable performance across all evaluation metrics. However, once the noise ratio exceeds 0.5, a steep decline in both sensitivity and F1 score is evident. This indicates a threshold effect, where a certain level of noise saturation leads to a tipping point beyond which the model's ability to accurately segment vessels deteriorates rapidly.

This observation is particularly relevant for practical applications, as it suggests that the U-Net can tolerate a moderate amount of noisy labels without significant loss in performance. However, when noise becomes predominant, the segmentation results are severely compromised. Thus, maintaining a low noise ratio in training data is crucial for achieving reliable model performance.

### 6.3 Significance of cleaning data

The results from Table 8 highlight the substantial benefits of data cleaning. In all cases, training on noise-free data produced better segmentation results compared to training on noisy data, even when the noisy data set was larger. This underscores the importance of data quality over quantity in machine learning tasks. The presence of noise not only hampers the model's learning process but also introduces biases that degrade its generalization ability.

The experiment further demonstrates that even minimal noise can adversely impact model performance, emphasizing the necessity for meticulous data preparation. Removing noise from the training data significantly improved both sensitivity and F1 score, illustrating that clean data leads to more accurate and reliable models. This finding is crucial for medical image analysis, where high precision and sensitivity are imperative for detecting subtle anatomical structures and pathological changes.

### 6.4 Implications for future work

Our study suggests several directions for future research. First, exploring more sophisticated noise-handling techniques, such as noise-robust loss functions or advanced data augmentation methods, could further enhance model robustness. Additionally, developing automated methods for noise detection and data cleaning could streamline the preprocessing pipeline, ensuring high-quality input data for training.

In conclusion, our experiments demonstrate the critical impact of noisy labels on retinal vessel segmentation and highlight the importance of data quality in medical imaging tasks. By carefully managing noise levels and ratios and employing effective data cleaning strategies, we can significantly improve model performance and reliability, ultimately contributing to better diagnostic outcomes in clinical practice.

## 7 Conclusion

This work addresses a largely overlooked problem in retinal vessel segmentation: positional annotation noise in manual labels. Prior retinal studies emphasize data quantity and quality, yet rarely model or measure the impact of positional label distortions on thin-vessel topology. We model realistic positional distortions, quantify performance sensitivity across noise levels and ratios, and introduce a lightweight few-shot annotation-correction framework that improves robustness with minimal labeling effort. On the analysis of the DRIVE dataset, increasing λ from 0 to 0.8 reduces sensitivity from 0.7758 to 0.4255 and F1 from 0.8170 to 0.5746. The method delivers strong generalization on CHASE_DB1 with an Accuracy of 96.51, AUC of 98.01, F1 of 83.55, and on STARE with an Accuracy of 97.54, AUC of 98.45, F1 of 83.11.

In future work, we will test more datasets and advanced segmentation networks in noisy environments by using our quantitative method, and give more general conclusions about the relationship between noise ratio and segmentation performance. Besides, we plan to add a new mechanism to the model that identifies capillaries so that the small vessels are trained separately. We will also propose a method to automatically identify and clean the noise.

## Data Availability

The original contributions presented in the study are included in the article/supplementary material, further inquiries can be directed to the corresponding author.

## References

[1] Abràmoff MD Garvin MK Sonka M. Retinal imaging and image analysis. IEEE Rev Biomed Eng. (2010) 3:169–208. doi: 10.1109/RBME.2010.2084567 22275207 PMC3131209

[2] Patton N Aslam TM MacGillivray T Deary IJ Dhillon B Eikelboom RH . Retinal image analysis: concepts, applications and potential. Prog Retin Eye Res. (2006) 25:99–127. doi: 10.1016/j.preteyeres.2005.07.001 16154379

[3] LeCun Y Bengio Y Hinton G. Deep learning. Nature. (2015) 521:436–44. doi: 10.1038/nature14539 26017442

[4] Niemeijer M Staal JJ van Ginneken B Loog M Abramoff MD. Comparative study of retinal vessel segmentation methods on a new publicly available database. Proc SPIE - Int Soc Opt Eng. (2004) 5370:648–56. doi: 10.1117/12.535349

[5] Hoover A Kouznetsova V Goldbaum M. Locating blood vessels in retinal images by piecewise threshold probing of a matched filter response. IEEE Trans Med Imaging. (2000) 19:203–10. doi: 10.1109/42.845178 10875704

[6] Owen CG Rudnicka AR Mullen R Barman SA Wormald R Cook DG . Measuring retinal vessel tortuosity in 20 infants with and without retinopathy of prematurity. Investig Ophthalmol Vis Sci. (2009) 50:1492–9. doi: 10.1167/iovs.08-3018 19324866

[7] Shen D Wu G Suk HI. Deep learning in medical image analysis. Annu Rev Biomed Eng. (2017) 19:221–48. doi: 10.1146/annurev-bioeng-071516-044442 28301734 PMC5479722

[8] Xiao X Lian S Luo Z Li S. Weighted res-unet for high-quality retina vessel segmentation. In: 2018 9th International Conference On Information Technology in Medicine and Education (ITME). Hangzhou: IEEE (2018). p. 327–31. doi: 10.1109/ITME.2018.00080

[9] Zhang C Bengio S Hardt M Recht B Vinyals O. Understanding deep learning (still) requires rethinking generalization. Commun ACM. (2021) 64:107–15. doi: 10.1145/3446776

[10] Jiang L Zhou Z Leung T Li LJ Fei-Fei L. Mentornet: learning data-driven curriculum for very deep neural networks on corrupted labels. In: International Conference on Machine Learning. Stockholm: PMLR (2018). p. 2304–13.

[11] Han B Yao Q Yu X Niu G Xu M Hu W . Co-teaching: robust training of deep neural networks with extremely noisy labels. arXiv preprint arXiv:180406872. (2018).

[12] Patrini G Rozza A Krishna Menon A Nock R Qu L. Making deep neural networks robust to label noise: a loss correction approach. In: Proceedings of the IEEE Conference on Computer Vision and Pattern Recognition. Honolulu, HI: IEEE (2017). p. 1944–52. doi: 10.1109/CVPR.2017.240

[13] Zhang T Yu L Hu N Lv S Gu S. Robust medical image segmentation from non-expert annotations with tri-network. In: I*nternational Conference on Medical Image Computing and Computer-Assisted Intervention*. Springer (2020). p. 249–58. doi: 10.1007/978-3-030-59719-1_25

[14] Dieleman S Willett KW Dambre J. Rotation-invariant convolutional neural networks for galaxy morphology prediction. Mon Not R Astron Soc. (2015) 450:1441–59. doi: 10.1093/mnras/stv632

[15] Krizhevsky A Sutskever I Hinton GE. Imagenet classification with deep convolutional neural networks. Adv Neural Inf Process Syst. (2012) 25:1097–105.

[16] Long J Shelhamer E Darrell T. Fully convolutional networks for semantic segmentation. In: Proceedings of the IEEE Conference on Computer Vision and Pattern Recognition. Boston, MA: IEEE (2015). p. 3431–40. doi: 10.1109/CVPR.2015.7298965 27244717

[17] Ronneberger O Fischer P Brox T. U-net: Convolutional networks for biomedical image segmentation. In: International Conference on Medical Image Computing and Computer-Assisted Intervention. Cham: Springer (2015). p. 234–41. doi: 10.1007/978-3-319-24574-4_28

[18] Badrinarayanan V Kendall A Cipolla R. Segnet: a deep convolutional encoder-decoder architecture for image segmentation. IEEE Trans Pattern Anal Mach Intell. (2017) 39:2481–95. doi: 10.1109/TPAMI.2016.2644615 28060704

[19] Alom MZ Hasan M Yakopcic C Taha TM Asari VK. recurrent residual convolutional neural network based on U-Net (R2U-Net) for medical image segmentation. In: NAECON 2018 - IEEE National Aerospace and Electronics Conference. Dayton, OH: IEEE (2018). doi: 10.1109/NAECON.2018.8556686

[20] Wang W Zhong J Wu H Wen Z Qin J. Rvseg-net: an efficient feature pyramid cascade network for retinal vessel segmentation. In: International Conference on Medical Image Computing and Computer-Assisted Intervention. Cham: Springer; (2020). p. 796–805. doi: 10.1007/978-3-030-59722-1_77

[21] Wang D Hu G Lyu C. Frnet: an end-to-end feature refinement neural network for medical image segmentation. Vis Comput. (2021) 37:1101–12. doi: 10.1007/s00371-020-01855-z

[22] Jin Q Meng Z Pham TD Chen Q Wei L Su R. DUNet: a deformable network for retinal vessel segmentation. Knowl-Based Syst. (2019) 178:149–62. doi: 10.1016/j.knosys.2019.04.025

[23] Liu C Gu P Xiao Z. Multiscale U-net with spatial positional attention for retinal vessel segmentation. J Healthc Eng. (2022) 2022:5188362. doi: 10.1155/2022/5188362 35047151 PMC8763561

[24] Brancati N Frucci M Gragnaniello D Riccio D. Retinal vessels segmentation based on a convolutional neural network. In: Iberoamerican Congress on Pattern Recognition. Cham: Springer (2017). p. 119–26. doi: 10.1007/978-3-319-75193-1_15

[25] Wang C Zhao Z Ren Q Xu Y Yu Y. Dense U-net based on patch-based learning for retinal vessel segmentation. Entropy. (2019) 21:168. doi: 10.3390/e21020168 33266884 PMC7514650

[26] Oliveira A Pereira S Silva CA. Retinal vessel segmentation based on fully convolutional neural networks. Expert Syst Appl. (2018) 112:229–42. doi: 10.1016/j.eswa.2018.06.034

[27] Oliveira A Pereira S Silva CA. Augmenting data when training a cnn for retinal vessel segmentation: how to warp? In: 2017 IEEE 5th Portuguese Meeting on Bioengineering (ENBENG). Coimbra: IEEE (2017). p. 1–4. doi: 10.1109/ENBENG.2017.7889443

[28] Dgani Y Greenspan H Goldberger J. Training a neural network based on unreliable human annotation of medical images. In: 2018 IEEE 15th International Symposium on Biomedical Imaging (ISBI 2018). Washington, DC: IEEE (2018). p. 39–42. doi: 10.1109/ISBI.2018.8363518

[29] Xue C Dou Q Shi X Chen H Heng PA. Robust learning at noisy labeled medical images: applied to skin lesion classification. In: 2019 IEEE 16th International Symposium on Biomedical Imaging (ISBI 2019). Venice: IEEE (2019). p. 1280–83. doi: 10.1109/ISBI.2019.8759203

[30] Liu J Li R Sun C. Co-correcting: noise-tolerant medical image classification via mutual label correction. IEEE Trans Med Imaging. (2021) 40:3580–92. doi: 10.1109/TMI.2021.3091178 34152981

[31] Shu Y Wu X Li W. Lvc-net: Medical image segmentation with noisy label based on local visual cues. In: International Conference on Medical Image Computing and Computer-Assisted Intervention. Cham: Springer (2019). p. 558–66. doi: 10.1007/978-3-030-32226-7_62

[32] Zhou Y Yu H Shi H. Study group learning: Improving retinal vessel segmentation trained with noisy labels. arXiv preprint arXiv:210303451. (2021). doi: 10.1007/978-3-030-87193-2_6

[33] Pizer SM Amburn EP Austin JD Cromartie R Geselowitz A Greer T . Adaptive histogram equalization and its variations. Comput Vis Graph Image Process. (1987) 39:355–68. doi: 10.1016/S0734-189X(87)80186-X

[34] Singnoo J Finlayson GD. Understanding the gamma adjustment of images. Color Imaging Conf . (2010) 2010:134–9. doi: 10.2352/CIC.2010.18.1.art00024

[35] Liu Z. Retinal vessel segmentation based on fully convolutional networks. arXiv preprint arXiv:191109915. (2019). doi: 10.48550/arXiv.1911.09915

[36] Srivastava N Hinton G Krizhevsky A Sutskever I Salakhutdinov R. Dropout: a simple way to prevent neural networks from overfitting. J Mach Learn Res. (2014) 15:1929–58.

[37] Staal J. Abràmoff MD, Niemeijer M, Viergever MA, van Ginneken B. Ridge-based vessel segmentation in color images of the retina. IEEE Trans Med Imaging. (2004) 23:501–9. doi: 10.1109/TMI.2004.825627 15084075

[38] Fraz MM Remagnino P Hoppe A Uyyanonvara B Rudnicka AR Owen CG . An ensemble classification-based approach applied to retinal blood vessel segmentation. IEEE Trans Biomed Eng. (2012) 59:2538–48. doi: 10.1109/TBME.2012.2205687 22736688

[39] Zhou Z Rahman Siddiquee MM Tajbakhsh N Liang J. Unet++: A nested u-net architecture for medical image segmentation. In: International Workshop on Deep Learning in Medical Image Analysis. Cham: Springer (2018). p. 3–11. doi: 10.1007/978-3-030-00889-5_1 PMC7329239 32613207

[40] Oktay O Schlemper J Folgoc LL Lee M Heinrich M Misawa K . Attention u-net: Learning where to look for the pancreas. arXiv preprint arXiv:180403999. (2018). doi: 10.48550/arXiv.1804.03999

[41] Wang Z Voiculescu I. Dealing with unreliable annotations: a noise-robust network for semantic segmentation through a transformer-improved encoder and convolution decoder. Appl Sci. (2023) 13:7966. doi: 10.3390/app13137966

[42] Kato S Hotta K. Adaptive t-vMF dice loss: an effective expansion of dice loss for medical image segmentation. Comput Biol Med. (2024) 168:107695. doi: 10.1016/j.compbiomed.2023.107695 38061152

[43] Gonzalez-Jimenez A Lionetti S Gottfrois P Gröger F Navarini A Pouly M. Robust t-loss for medical image segmentation. Med Image Anal. (2025) 105:103735. doi: 10.1016/j.media.2025.103735 40743711

[44] Zeng Z Liu J Huang X Luo K Yuan X Zhu Y. Efficient retinal vessel segmentation with 78K parameters. J Imag. (2025) 11:306. doi: 10.3390/jimaging11090306 41003356 PMC12470885

[45] Seo S Yoon H Kim S Lee J. Full-scale representation guided network for retinal vessel segmentation. arXiv preprint arXiv:250118921. (2025). doi: 10.1186/s12880-025-02021-4 41272503 PMC12639801

